# Intracerebral Hemorrhage Prognosis Classification via Joint-Attention Cross-Modal Network

**DOI:** 10.3390/brainsci14060618

**Published:** 2024-06-20

**Authors:** Manli Xu, Xianjun Fu, Hui Jin, Xinlei Yu, Gang Xu, Zishuo Ma, Cheng Pan, Bo Liu

**Affiliations:** 1 The Second Affiliated Hospital of Zhejiang Chinese Medical University, Hangzhou 310053, China; 20114012@zcmu.edu.cn; 2 School of Artificial Intelligence, Zhejiang College of Security Technology, Wenzhou 325016, China; fuxianjun@zjcst.edu.cn; 3 School of Computer, Hangzhou Dianzi University, Hangzhou 310018, China; 22050927@hdu.edu.cn (H.J.); 21010237@hdu.edu.cn (X.Y.); 4 International Business School, Jinan University, Zhuhai 510632, China; mzs123@stu2022.jnu.edu.cn; 5 School of General Education, Sanda University, Shanghai 201209, China; panc@sandau.edu.cn; 6 The 39th Research Institute of China Electronics Technology Group Corporation, Xi’an 710065, China; liub_o@stu.xjtu.edu.cn; 7 Key Laboratory of Antenna and Control Technology of Shanxi Province, Xi’an 710068, China; 8 School of Management, Xi’an Jiaotong University, Xi’an 710049, China

**Keywords:** intracerebral hemorrhage, clinical information, cross-modal fusion, joint-attention mechanism, cross-modal loss

## Abstract

Intracerebral hemorrhage (ICH) is a critical condition characterized by a high prevalence, substantial mortality rates, and unpredictable clinical outcomes, which results in a serious threat to human health. Improving the timeliness and accuracy of prognosis assessment is crucial to minimizing mortality and long-term disability associated with ICH. Due to the complexity of ICH, the diagnosis of ICH in clinical practice heavily relies on the professional expertise and clinical experience of physicians. Traditional prognostic methods largely depend on the specialized knowledge and subjective judgment of healthcare professionals. Meanwhile, existing artificial intelligence (AI) methodologies, which predominantly utilize features derived from computed tomography (CT) scans, fall short of capturing the multifaceted nature of ICH. Although existing methods are capable of integrating clinical information and CT images for prognosis, the effectiveness of this fusion process still requires improvement. To surmount these limitations, the present study introduces a novel AI framework, termed the ICH Network (ICH-Net), which employs a joint-attention cross-modal network to synergize clinical textual data with CT imaging features. The architecture of ICH-Net consists of three integral components: the Feature Extraction Module, which processes and abstracts salient characteristics from the clinical and imaging data, the Feature Fusion Module, which amalgamates the diverse data streams, and the Classification Module, which interprets the fused features to deliver prognostic predictions. Our evaluation, conducted through a rigorous five-fold cross-validation process, demonstrates that ICH-Net achieves a commendable accuracy of up to 87.77%, outperforming other state-of-the-art methods detailed within our research. This evidence underscores the potential of ICH-Net as a formidable tool in prognosticating ICH, promising a significant advancement in clinical decision-making and patient care.

## 1. Introduction

Intracerebral hemorrhage (ICH) constitutes a severe threat to human health, ac-counting for 20% to 30% of all stroke cases. As a critical cerebrovascular condition, ICH is characterized by its complex etiologies and heterogeneous clinical presentations. Within the first 30 days post-onset, the mortality rates for ICH patients remain alarmingly high, ranging from 35% to 52% [1]. Additionally, a prospective observational cohort study demonstrated a cumulative recurrence rate of 6.1% within the first year, increasing to 7.9% by the fifth year following a lobar hemorrhage [2]. Furthermore, survivors of ICH often face the prospect of enduring long-term disabilities, epilepsy, blood clotting, vision, or vascular issues [3]. Considering the notable incidence, disability, and mortality rates associated with ICH, the urgency of timely and precise diagnostic processes cannot be overstated [4].

Historically, the diagnosis of ICH has relied upon the professional understanding and empirical knowledge of physicians, who interpret computed tomography (CT) scans by examining parameters such as the location, volume, and distinctive texture characteristics of the hemorrhagic site, in conjunction with the Glasgow Coma Scale (GCS) score [5]. This conventional method is inherently subjective, heavily dependent on the clinician’s expertise, and can be resource-intensive.

To mitigate these issues, earlier research adopted machine learning techniques with promising outcomes. However, the potential for further enhancement remains. The burgeoning field of artificial intelligence (AI) has heralded significant advancements in medical imaging technology, thereby enhancing the comprehensiveness of imaging data available for clinical use. This innovation plays an increasingly pivotal role in facilitating disease screening, informing treatment planning, and assessing prognostic outcomes. Biomedical images are particularly informative, as they encapsulate crucial information reflecting underlying pathophysiological changes. CT, especially the widely utilized and straightforward non-contrast-enhanced CT, is instrumental in the diagnosis and management of ICH and its potential complication, hematoma expansion. Diagnostic signs detectable on non-contrast CT, such as the black hole sign [6], mixed-density sign, low-density areas, and the island sign, hold clinical significance for predicting hematoma growth. Nevertheless, the interpretation of these imaging features hinges on the expertise of well-trained clinicians and is subject to the limitations of an individual reader’s experience and subjective judgment, which often results in low sensitivity. AI models offer a solution to surmount these challenges. By mitigating the impact of subjective biases during the analysis, AI can provide more precise and reproducible assessments. In addition, the integration of AI into medical imaging analysis has the potential to augment the objective evaluation of signs, such as those indicative of hematoma expansion in ICH, to improve the quality of patient care and outcomes.

To refine diagnostic accuracy, contemporary AI methods have embraced more sophisticated algorithms. In the medical domain, AI has demonstrated notable successes in diagnosing conditions, such as breast cancer [7], prostate cancer [8], intracranial hematoma [9], and pleural effusion [10]. In the specific context of ICH, cross-modal methods that leverage comprehensive datasets have become increasingly relevant. Recent advancements in deep learning models have been adopted to effectively boost ICH diagnosis. For instance, Wang et al. [11] proposed a data fusion framework based on convolutional neural networks (CNNs) for early prediction of hematoma expansion. Likewise, del Barrio et al. [12] presented a deep learning model based on CNN for prognosis prediction after ICH. Also, the current methods include variational autoencoder (VAE) [13,14,15,16] and generative adversarial networks (GANs) [17]. In the current landscape, image-based methodologies [18] and multi-task strategies [19] have been employed in this domain, producing commendable outcomes. Specifically, the Res-Inc-LGBM model [20], a cross-modal technique that extracts information from two distinct modalities within CT imagery, has demonstrated promising results. Nevertheless, this model did not utilize clinical data to further enhance its efficacy. In addition, the UniMiSS framework [21] represents an innovative approach by incorporating an extensive array of 2D medical images into a 3D self-supervised learning paradigm, thereby addressing the limitation imposed by the paucity of 3D datasets, such as those obtained from CT scans. Additionally, GCS-ICH-Net [22] has improved performance by employing a self-attention mechanism to integrate imaging data with domain knowledge. However, existing methodologies have yet to implement effective fusion mechanisms.

To rectify these deficiencies, this article puts forth a novel approach with the following advantages:

(1) We introduce a cross-modal loss function that accounts for the intrinsic correlation between the disparate data modalities.
(2) We incorporate clinical data to enrich the model’s comprehension and enhance ICH prognosis accuracy.
(3) Our fusion model incorporates a joint-attention mechanism, effectively facilitating the extraction of more salient and comprehensive fusion features.

This innovative strategy promises to enhance the precision of ICH diagnosis, thereby facilitating more effective patient management and improving clinical outcomes. In our study, we incorporated spontaneous ICH into our dataset, explicitly excluding cases with causes such as arteriovenous malformations, cerebral aneurysms, traumatic brain injury, brain tumors, and cerebral infarctions.

## 2. Materials and Methods

### 2.1. Problem Formalization

Within the specified dataset comprising patients diagnosed with ICH, each patient’s record encompasses clinical data alongside one or more CT slices. The objective of this study is to develop a predictive model that, when trained on the designated training set, can process the provided inputs and yield outputs that closely align with the target la-bels. Upon evaluation using the test set, the model demonstrated commendable performance.

### 2.2. Patient Population

A retrospective study was conducted on a cohort of 294 patients who were admitted to our hospital with spontaneous ICH from August 2013 to May 2021 and completed the prescribed treatment regimen. The study received approval from the hospital’s ethics committee and informed verbal consent was obtained from all participants. The inclusion criteria for the data were as follows: (1) diagnosis of spontaneous ICH confirmed, (2) completion of plain CT scans within 24 h after the cessation of bleeding, (3) availability of complete GCS scores at admission, (4) prognostic data based on the Glasgow Outcome Scale (GOS) at discharge, and (5) comprehensive clinical information, including age, gender, and location of hemorrhage, among other variables. Patients presenting with secondary ICH resulting from arteriovenous malformation, cerebral aneurysm, traumatic brain injury, brain tumor, or cerebral infarction were excluded from the study. Regarding imaging equipment, the study utilized a Philips Brilliance 16-slice CT scanner and a Toshiba Aquilion ONE 320-slice CT scanner. The scans were conducted with a slice thickness of 6 mm and a matrix size of 512 × 512, which has voxel sizes in millimeters (0.488, 0.488, and 6).

### 2.3. Data Acquisition

Our proprietary dataset comprised 294 clinical cases obtained from our partner hospital, representing a balanced collection, with 149 cases classified as having positive outcomes and 145 cases with negative outcomes. The prognostic labels for these datasets were determined by three neurosurgeons, one with a senior professional title, one with an intermediate professional title, and one with a junior professional title. Prognosis was predicted using a double-blind method based on two approaches: utilizing image features alone and combining image features with GCS scoring information to assess the prognosis of enrolled ICH cases. Patient demographics and clinical characteristics were extracted from the electronic medical record system, encompassing variables such as gender, age, CT scan acquisition time, length of hospital stay, GCS score, treatment methodology, and the location and volume of the hemorrhage. If the patient has a good prognosis, they are unlikely to experience any other concurrent symptoms following treatment. Conversely, a patient with a poor prognosis may develop sequelae in the later stages of treatment, such as hemiplegia, language disorders, and decreased muscle strength. Prognosis was stratified based on the GOS, with a GOS score of ≥4 indicating a good prognosis, and a GOS score of ≤3 indicating a poor prognosis. Additionally, a GOS of 1–5 corresponds to outcomes ranging from death, vegetative state, severe disability, mild disability, to return to normal life.

In our dataset, the good prognosis group included 149 patients, 109 males and 40 females, with an age range of 29–88 years and a mean age of 53.85 years. Their hospitalization period varied from 3 to 104 days, with an average stay of 19.40 days. Treatment approaches varied, with 119 patients receiving conservative management in the internal medicine department and 25 undergoing surgical interventions. Hemorrhage locations were distributed as follows: 48 cases in the basal ganglia, 29 in the thalamus, 5 in the external capsule, 19 in the cerebral lobes, 16 in the brainstem, 14 in the cerebellum, 6 in the ventricles, 5 in multiple regions, and 32 with secondary ventricular involvement. Conversely, in the poor prognosis group, there were 145 individuals, comprising 108 males and 37 females, with an age range of 29 to 90 years and a mean age of 54.16 years. The duration of hospital stays ranged from 1 to 388 days, with an average of 39.63 days. In terms of treatment, 67 patients received conservative care within internal medicine, while 75 underwent surgical procedures. The distribution of hemorrhage locations included 76 cases in the basal ganglia, 18 in the thalamus, 6 in the external capsule, 12 in the cerebral lobes, 13 in the brainstem, 2 in the cerebellum, 1 in the ventricles, 10 spanning multiple regions, and 49 associated with secondary ventricular bleeding. Although no universally accepted GCS threshold exists for predicting the prognosis in ICH patients, it is generally held by clinicians that a GCS score of 9 or above is predictive of a more favorable outcome.

### 2.4. ICH-Net Architecture

Enhancing the accuracy of prognosis classification for ICH can significantly reduce mortality and disability risks. However, current methods fail to efficiently integrate clinical and imaging data for prognosis classification, thereby limiting their effectiveness in aiding accurate diagnosis by medical professionals. To address the challenge of integrating clinical and imaging data and to enhance prognosis classification accuracy, we proposed ICH-Net, a novel network framework.

**Figure 1:**
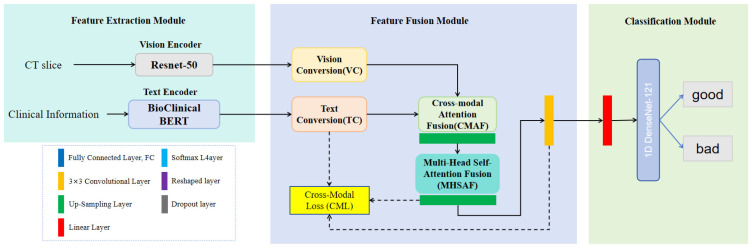
The diagram illustrates the architecture of ICH-Net, which comprises a Feature Extraction Module, a Feature Fusion Module, and a Classification Module, in sequential order. Ultimately, it outputs the final results.

The architecture of our ICH-Net is depicted in Figure 1 and is composed of three sequential components: the Feature Extraction Module, the Feature Fusion Module, and the Classification Module. Notably, the Feature Fusion Module employs a cross-modal attention mechanism to fully account for the intrinsic relationships between the different modalities, thereby enabling targeted and pertinent feature integration within the network.

Clinicians utilize imaging data and clinical texts to comprehensively assess prognosis. Clinical text information, such as GCS, offers domain-specific knowledge that aids in prognostic evaluation. Additionally, factors such as age, gender, and other demographic information have distinct impacts on ICH outcomes. Consequently, we designed various modules to extract both image and text features. In the Feature Extraction Module, we utilized two distinct encoders: $f^{t}$ and $f^{v}$, to obtain the textual representation and the visual representation, respectively. For text encoding, we leveraged the pre-trained BioClinicalBERT model [23], while for visual encoding, we employed the pre-trained 2D ResNet50 architecture [24].

Within the Feature Fusion Module, the textual representation, $f^{t}$, and the visual representation, $f^{v}$, are processed through a Text Conversion (TC) sub-module and a Vision Conversion (VC) sub-module, respectively. The outputs from these sub-modules, denoted as ${\hat{f}}^{t}$ and ${\hat{f}}^{v}$, were subsequently input into the Cross-Modal Attention Fusion (CMAF) and the Multi-Head Self-Attention Fusion (MHSAF). This step facilitated the effective integration of textual and visual representations. Finally, in the Classification Module, the fused representation was passed through our neural network, culminating in a classification task that discerns the prognostic outcomes.

### 2.5. The Detail Blocks

**Figure 2:**
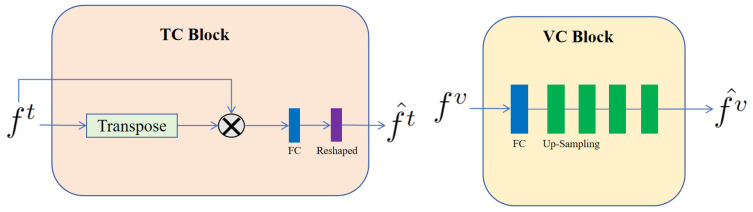
Architecture of the TC block and VC block. These blocks were designed to specifically process text and visual information, respectively. The symbol ⊗ stands for matrix multiplication.

TC block and VC block. In our architecture, the TC and VC blocks are integral to the efficacious fusion of textual and visual data. As depicted in Figure 2, the TC block began by calculating the product of the text representation, ${\hat{f}}^{t}$, and its transpose. This operation facilitated the modeling of associations between each word in the text representation and its counterparts, thereby capturing the semantic relationships and contextual nuances inherent within the text sequence. Leveraging an attention-based mechanism, the TC block enhanced the model’s comprehension of the textual input and fostered a more nuanced and information-rich representation during text data processing. Following this, the resulting matrix was subjected to a transformation via a Fully Connected (FC) layer. The FC layer was designed to discern nonlinear relationships within the input data, ultimately yielding output representations that are tailored to meet the specific demands of our task. The sequence concluded with the reshaping of this output to produce ${\hat{f}}^{t}$, which represents the refined final form of the text data, as processed by the TC blocks.

In contrast, within the VC block of our architecture, we handled the visual representation, ${\hat{f}}^{v}$, derived from the input visual data. To refine the integration of these visual features, the initial step involved passing them through a FC layer. This FC layer executed linear transformations on the visual representations, thereby remapping them into a representation space that was more apt for the ensuing analytical steps. Subsequent to the FC layer’s processing, the visual representations underwent a sequence of four up-sampling operations. These operations incrementally enhanced the resolution of the feature map, enabling a more detailed and precise capture of the structural nuances present in the input image. The amalgamation of these transformative components culminated in the output visual representation, ${\hat{f}}^{v}$, which embodied an effectively processed and integrated depiction of the visual feature set.

**Figure 3:**
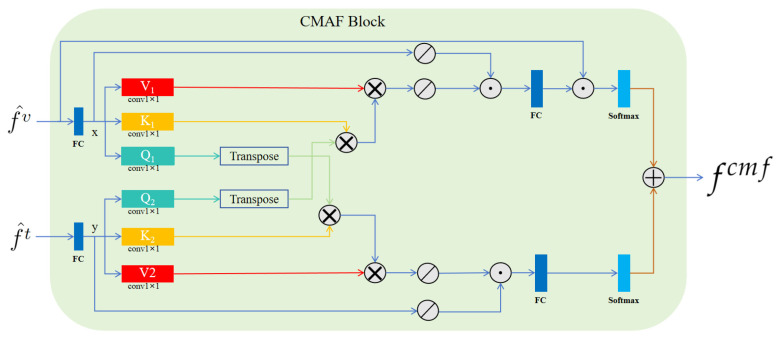
Architecture of the proposed CMAF block. Here, ⊗ represents matrix multiplication, ⊘ stands for SoftPool, ⊙ symbolizes matrix addition, and ⊕ signifies concatenation. In this block, ${\hat{f}}^{v}\;and\;{\hat{f}}^{t}$ are inputs, then $f^{cmf}$ is obtained as the output.

CMAF block. Inspired by the methodological framework of CMAFGAN [25], for simple notation, we denoted ${\hat{f}}^{t}$ and ${\hat{f}}^{v}\;$ as x and y, respectively. As shown in Figure 3, the CMAF block began with six 1 × 1 convolution layers applied to x and y. These layers transformed each input into three matrices—V1, K1, and Q1 for x, and V2, K2, and Q2 for y—with their associated weights represented by ω. The transformation can be formalized to yield the attention matrices, as follows:

$$\alpha_{j,i}=\frac{\exp\left(\omega^{Q1}x_{i}^{⊤}\cdot \omega^{K2}y_{j}\right)}{\sum_{i=1}^{n}\exp\left(\omega^{Q1}x_{i}^{⊤}\cdot \omega^{K2}y_{j}\right)},\;\beta_{j,i}=\frac{\exp\left(\omega^{Q2}y_{i}^{⊤}\cdot \omega^{K1}x_{j}\right)}{\sum_{j=1}^{m}\exp\left(\omega^{Q2}y_{i}^{⊤}\cdot \omega^{K1}x_{j}\right)}$$

where $\omega^{Q1}x_{i}^{⊤}$ signifies the linear transformation of the input $x_{i}$ via the weight matrix $\omega^{Q1}$ followed by transposition. Subsequently, as depicted in Figure 3, we obtained the final output representation, $f^{cmf}$, through these operations. Here, ${\hat{f}}^{cmf}$ is the representation of $f^{cmf}$ after passing through an up-sampling layer.

**Figure 4:**
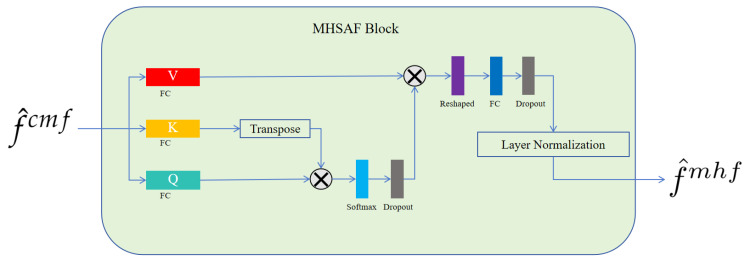
Detailed architecture of the proposed MHSAF block. The symbol ⊗ denotes matrix multiplication.

MHSAF block. As depicted in Figure 4, to enhance feature representation, our approach projected features onto three distinct subspaces, utilizing a trio of independent linear transformations, each governed by a unique weight matrix. Following this projection, self-attention computations were executed within each subspace to yield a set of output vectors. These vectors were subsequently concatenated, resulting in a comprehensive final output that captured the diversified interactions within the data. This method allowed for a more nuanced and multi-faceted analysis by capitalizing on the strengths of multiple representational spaces.

The merit of this methodology lies in its capacity to process features with enhanced granularity across disparate subspaces while concurrently conducting self-attention computations within each individual subspace. This approach enables the model to more adeptly discern the inter-feature relationships from varied perspectives. By carrying out attention-based calculations within each designated subspace, the model gains a more profound comprehension of the dependencies among features, which allows for the extraction of richer and more precise information. Ultimately, this refined understanding contributes to the improvement of the model’s final predictive accuracy.

Furthermore, by concatenating multiple output vectors from within each subspace to construct the final output, we enable the effective integration of information across each subspace. This process yields a more holistic and nuanced representation, thereby augmenting the model’s expressive capabilities and enhancing its predictive accuracy. In essence, by distributing features across various subspaces and executing self-attention computations within these discrete domains, we capitalize on the inter-feature relationships. This strategy not only facilitates the extraction of richer and more precise information but also substantially elevates the predictive efficacy of the model.

### 2.6. Loss Function

We utilized a composite loss function termed Cross-Modal Fusion (CMF) Loss, which comprises three components: Intra-Modality and Inter-Modality Alignment (IMIMA) loss, Similarity Distribution Matching (SDM) loss, and Masked Language Modeling (MLM) loss.

IMIMA Loss. We deployed four principal loss terms, namely, Text-to-Text (t2t), Vision-to-Vision (v2v), Vision-to-Text (v2t), and Text-to-Vision (t2v). We designated the negative sample set for a given sample as N. The formulation for each loss component is as follows:

$$L_{\mathrm{intra}/\mathrm{inter}}^{A2B}=-\log\frac{\delta \left(f^{A},f^{B}\right)}{\delta \left(f^{A},f^{B}\right)+\sum_{f_{k}\in N}\delta \left(f^{A},f_{k}^{B}\right)},$$

where the pairwise similarity measure $\delta \left(a,b\right)=\exp\left(a^{⊤}b\right)$.

To encapsulate the total loss, we aggregated these terms to define the IMIMA loss:

$$L_{IMAMA}=L_{intra}^{t2t}+L_{intra}^{v2v}+L_{inter}^{t2v}+L_{inter}^{v2t}.$$

SDM Loss. We incorporated the SDM loss, as delineated by Jiang et al. [26], to quantify the discrepancy between the predicted similarity distribution and the ground-truth similarity distribution produced by the model. The computation of this loss leverages the Kullback–Leibler (KL) divergence, encompassing bi-directional components: Visual-to-Text (v2t) and Text-to-Visual (t2v). The formulation of this loss function was meticulously designed to steer the model toward a more accurate alignment of similarity distributions between visual and textual representations of the input data. Such alignment is instrumental in amplifying the model’s performance across cross-modal tasks. The v2t loss is defined as follows:

$$L_{t2v}=KL\left(p_{i}|q_{i}\right)=\frac{1}{n}\sum_{i=1}^{n}\sum_{j=1}^{n}p_{i,j}\log\left(\frac{p_{i,j}}{q_{i,j}}\right),$$

where $q_{i}$ represents the true probability distribution of matches for the i-th sample, and $p_{i}$ denotes the SoftMax-normalized cosine similarity scores. In this context, $p_{i,j}$ is the predicted probability that the i-th sample corresponds to the j-th category, and $q_{i,j}$ is the ground-truth probability.

Consequently, the cumulative SDM loss is the sum of the v2t and t2v losses, calculated by:

$$L_{SDM\;}=L_{v2t\;}+L_{t2v\;}.$$

MLM Loss. Drawing on the architectural principles of the BERT model, we devised a novel method. Initially, we obscured select words within the input sequence using a specialized masking token. Subsequently, the model’s predicted probability distribution for these masked positions was contrasted with the actual labels to assess the model’s predictive bias. This bias was quantified and integrated into the loss function as a facet of the training process. The intent behind this methodology was to aid the model in attaining a more profound comprehension of the context enveloping the input sequence. By diminishing the disparity between the predicted and true labels, we aimed to bolster the model’s overall performance.

Ultimately, our CMF loss function is a weighted sum of the individual loss components. Each loss term was assigned a corresponding weight, reflected in the final formulation. The comprehensive loss function is expressed as follows:

$$L_{CMF\;}=L_{IMAMA\;}+{\alpha L}_{SDM}+\beta L_{MLM},$$

where α and β are hyperparameters that balance the contributions of the SDM and MLM losses, respectively, alongside the IMIMA loss.

## 3. Results

### 3.1. Data Pretreatment

In the preprocessing of the imaging data, we employed multi-threshold segmentation and connected component analysis to delineate the regions of interest, thereby mitigating interference from non-brain-tissue areas. Threshold segmentation was applied to discriminate bone structures from other tissues, effectively isolating the hemorrhagic zones and preserving normal brain parenchyma. To further enhance precision and eliminate extraneous noise, connected component analysis was performed in a batch-processing manner. The objective was to ensure that the final image comprised solely of pertinent brain tissue regions; specifically, the hemorrhagic sites, gray matter, and intact brain tissue. This meticulous approach to image preprocessing is critical for the accuracy of subsequent analyses.

Regarding the text data preprocessing, we extracted key variables, such as age, gender, time from onset to CT, hospital stay, GCS score, treatment method, and physician’s diagnosis, from the clinical dataset. Then, each variable was individually processed through a word segmentation tool integrated within the pre-trained Bio-ClinicalBERT model. This process facilitated the conversion of the textual data into a tensor representation to be suitable for subsequent computational analysis.

### 3.2. Experiments

To demonstrate the superiority and validate the robustness of our methodology, we executed a comprehensive suite of experiments, encompassing both comparative and ablation studies. Throughout the training phase, we meticulously optimized hyperparameters. Specifically, we set the learning rate at 0.0001, determined the number of training epochs to be 300, and established the batch size at 128. These configurations were carefully chosen to balance computational efficiency with model performance. The libraries used in our experiment included but were not limited to torch = 1.21.1 + cu116 and torch vision = 0.13.1 + cu116. And the code is available at https://github.com/YU-deep/ICH-2D, accessed on 18 June 2024.

#### 3.2.1. Comparative Experiments

As detailed in Table 1, our comparative study benchmarked our algorithm against the leading contemporary methodologies, which span purely 3D, purely 2D, and hybrid 2D + 3D approaches. The results underscored our method’s superiority across all evaluated metrics, thereby confirming its efficacy. Specifically, when juxtaposed with the best-performing metrics of alternative methods, our approach exhibited an increment of 2.35% in accuracy (ACC), a 0.13% rise in recall, and a 0.0027 enhancement in the area under the receiver operating characteristic curve (AUC). Despite employing a 2D-based framework, these results clearly demonstrated that our method’s overall performance surpassed that of both existing 3D and 2D techniques.

Based on the outcomes of our analysis, we posit that the observed superiority of our method can be attributed to the following factors:

(1) Multimodal Information Fusion: Our model incorporated a CMF loss function, which effectively harnessed the intrinsic correlations between various modalities. By synergistically integrating CT images with clinical data, our model achieved a more holistic understanding of the tasks at hand, consequently enhancing its overall performance.
(2) Feature Fusion Mechanism: Our CMAF module employed a cross-modal attention mechanism designed to extract salient and comprehensive fusion features. This method facilitated a more discerning aggregation of information from multiple sources, enhancing the representational power of the fused features.
(3) Utilization of Advanced Pre-trained Models: Our framework incorporated two distinct modules for feature extraction—a visual feature extraction module utilizing the ResNet50 model and a text feature extraction module employing the BioClinicalBERT model. These pre-trained models were instrumental in enhancing the capability of our system to extract more robust and nuanced features. By leveraging the extensive knowledge encoded within these pre-trained models, our approach achieved superior feature extraction performance.

#### 3.2.2. Ablation Experiment

As presented in Table 2, the Vision-Only approach exclusively processed visual data within the model, whereas the Text-Only approach was limited to textual information. The tabulated results elucidated that the cross-modal input strategy implemented in our ICH-Net significantly enhanced the performance, yielding an 11.18% increase in accuracy and a 0.0934 improvement in AUC compared to the Vision-Only method. These findings affirm the value of integrating clinical information to enhance diagnostic accuracy.

Our perspective posits that patient information extends beyond the confines of isolated modalities, such as CT images or clinical data; rather, there is an intrinsic interrelation between these two forms of information. A concurrent comprehension of both modalities can significantly augment the model’s proficiency in executing predictive tasks. By harnessing this synergistic understanding, our model was tailored to leverage the compounded insights gained from the integrated analysis of multimodal data, thereby enhancing the accuracy and efficacy of its predictions.

### 3.3. Visualization Analysis

**Figure 5:**
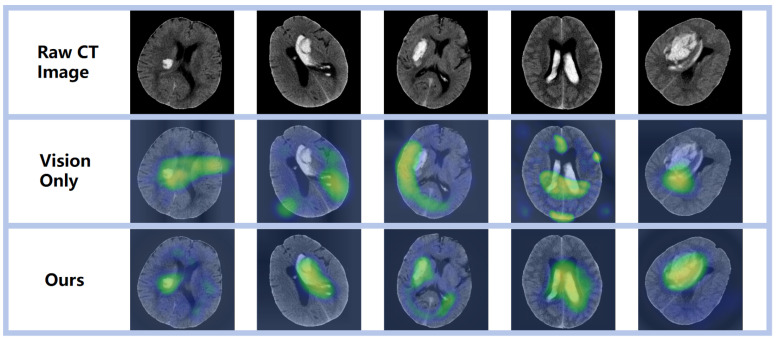
Visual representation of diverse prediction outcomes, emphasizing the activation zone of class association in the predicted network with a prominent red hue.

Through meticulous examination of the images within the test set and the delineation of regions of interest, we gained a refined understanding of the network’s recognition accuracy of areas afflicted by ICH. As depicted in Figure 5, the employed visualization technique served a dual purpose: it corroborated the model’s remarkable precision in localizing hemorrhagic sites and underscored its capacity for accurate hemorrhage localization predictions—both of which are pivotal for assessing the network’s predictive capabilities. Moreover, these visualization outcomes provided a more profound insight into the network’s cognitive mechanisms, specifically its process for identifying hemorrhagic areas within images. Such clarity presents an opportunity to refine and enhance network performance. By deciphering the pivotal features and patterns that the network relies on for decision-making, we can implement targeted adjustments and advancements, thereby augmenting its efficacy in recognizing ICH. Consequently, this thorough inspection and visualization approach yielded indispensable insights for model refinement and the optimization of predictive performance. It equipped us with a deeper comprehension of the complexity inherent in the tasks at hand and propelled the enhancement of our models’ predictive acumen.

## 4. Discussion

Our investigation spanned numerous datasets, yet we found that none offered concurrent public access to both imaging and clinical information. Our private dataset stood out as both comprehensive and reflective of real-world conditions. However, future applications to external datasets may influence the performance of our model. Thus, enhancing the model’s generalization capability remains a primary objective.

Within our methodological framework, segmentation techniques were not utilized. We posit that approaches eschewing segmentation might offer a more holistic consideration of a patient’s condition, encompassing clinical presentation, imaging findings, medical history, and other pertinent factors. Such an approach could potentially aid physicians in conducting a more thorough prognostic assessment of patients and formulating optimal treatment strategies. Nevertheless, it is acknowledged that both segmentation and non-segmentation methods have their respective merits and limitations, contingent on the clinical scenario.

Our study identified several pathways for further enhancement and outlined key areas for future research. Firstly, the dataset used was sourced from collaborating hospitals, which may limit its size and diversity, potentially reducing the effectiveness of our model when applied to datasets from different institutions or that include varied data types. Secondly, during feature extraction, we utilized pre-trained text and visual encoders. Should these models perform sub-optimally on certain tasks, the efficacy of our methodology could be compromised. Lastly, our model integrated CT imaging data and text, which has been demonstrated to influence prognosis classification outcomes, such as gender [27,28,29], early cognitive status [30], and the location and volume of the hemorrhage [31,32]. Therefore, any loss of information between modalities might impair the model’s performance. These identified limitations guide a roadmap for future improvements, including expanding the dataset’s scope and diversity, enhancing feature extraction techniques, and mitigating information loss across different modalities.

## 5. Conclusions

In current methodologies, the lack of effective fusion mechanisms has resulted in the suboptimal amalgamation of clinical data with CT imaging for the prognostic classification of ICH across different modalities. To address this shortfall, we proposed a pioneering framework named ICH-Net, which stands for joint-attention cross-modal network. ICH-Net comprises a Feature Extraction Module, a Feature Fusion Module, and a Classification Module. Additionally, ICH-Net incorporates a CMF loss function, which includes IMAMA loss, SDM loss, and MLM loss, to enhance modality alignment and improve the model’s interpretability with respect to the task at hand. Moreover, in the CMAF block, a cross-modal attention mechanism was employed to strategically focus on significant regions within the data.

Our empirical assessments, encompassing both comparative and ablation studies, have substantiated the efficacy of our proposed approach. In our future work, we plan to collect a more comprehensive dataset of clinical information to strengthen the model’s generalizability. Furthermore, we aim to extend the application of ICH-Net to a broader spectrum of tasks by embracing multi-modal and multi-task learning paradigms.

## Figures and Tables

**Table 1:** The comparative experiments comparing our method with other methods.

Methods	ACC (%)	Recall (%)	Precision (%)	AUC
DL-Based Method (3D) [12]	81.02	78.52	83.31	0.9141
Image-Based Method (2D) [18]	74.23	67.11	75.98	0.6933
Multi-Task Method (3D) [19]	85.42	79.86	**89.80**	0.8998
GCS-ICH-Net (2D) [22]	85.08	81.88	87.25	0.8590
UniMiSS (2D + 3D) [21]	82.03	78.52	87.59	0.8275
**ICH-Net** (**Our study**)	**87.77**	**82.01**	88.23	**0.9168**

Bold indicates the best, and underline is the second best.

**Table 2:** Ablation experiment on loss function.

Methods	ACC (%)	Recall (%)	Precision (%)	AUC
Vision-Only	76.59	73.10	80.84	0.8234
Text-Only	69.15	65.10	71.11	0.7534
**ICH-Net** (**Our study**)	**87.77**	**82.01**	**88.23**	**0.9168**

Bold indicates the best, and underline is the second best.

## Data Availability

Our data were obtained from collaborating hospitals and are not publicly available.
